# Supplementary material for: A Unique Platform to Study the Dynamic Process of Prion Formation
Source: Biochemistry. 2026 Aug 25;65(18):2841–50. doi: 10.1021/acs.biochem.6c00351 (PMC13584988; doi:10.1021/acs.biochem.6c00351)
Supplement: Supplementary file 1 [file bi6c00351_si_001.pdf]

Supporting Information for

A unique platform to study the dynamic process of prion formation

Jessica de Alcantara Ferreira[1], Daniel J. Walsh[1], Evelyn Turnbaugh[1], Jeremy H. Mills[2], and Surachai Supattapone[1,3,*]

Departments of Biochemistry[1] and Medicine[3], Geisel School of Medicine at Dartmouth, Hanover, New Hampshire 03755, USA

[2]School of Molecular Sciences and The Biodesign Center for Molecular Design and Biomimetics, Arizona State University

Tempe, AZ 85287, USA

[*]Corresponding author

Mailing address: Department of Biochemistry and Cell Biology, 7200 Vail Building,

Geisel School of Medicine and Dartmouth, Hanover, New Hampshire 03755, USA.

Phone (603) 646-5212. Email: supattapone@dartmouth.edu

Table of Contents

Figure S1. Expression of various 7-HCAA mutant PrP molecules in *E. coli*

Figure S2. Electrophoretic mobility of purified W99 7-HCAA recPrP

Figure S3. Mass spectrometric characterization of W99 7-HCAA recPrP

Figure S4. Circular dichroism (CD) spectrum of W99 7-HCAA recPrP and WT recPrP

Figure S5. Regional neuropathology of kiBV M109 PrP mice inoculated with W99 7-HCAA cofactor PrP^{Sc} and WT cofactor PrP^{Sc}

Figure S6. Time-dependent changes in the non-denatured/denatured fluorescence ratio during PrP^{Sc} formation.

Table S1. Integrated fluorescence intensity of PrP samples

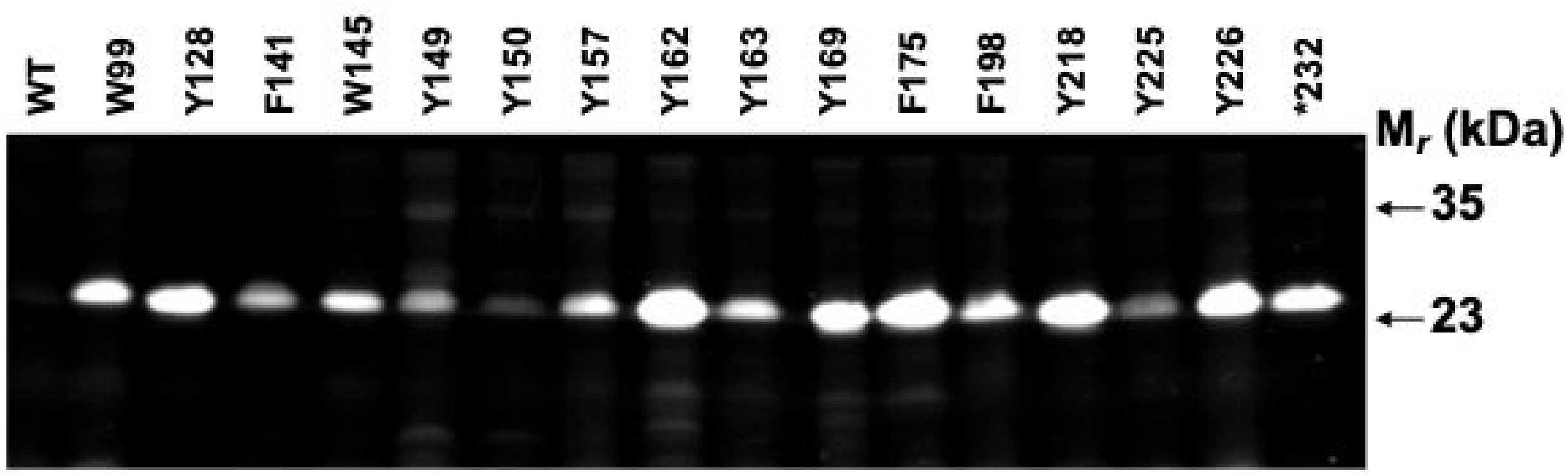

Figure S1: Expression of various 7-HCAA mutant PrP molecules in *E. coli*. Fluorescence-imaged SDS polyacrylamide gel of crude lysates prepared from bacteria expressing mutant PrP molecules with 7-HCAA substituted for various residues, as indicated. WT = control bacteria expressing wild-type PrP without 7-HCAA substitution. *232 = amber codon was inserted immediately after the final residue S232 to produce full-length PrP with 7-HCAA added to the C-terminus.

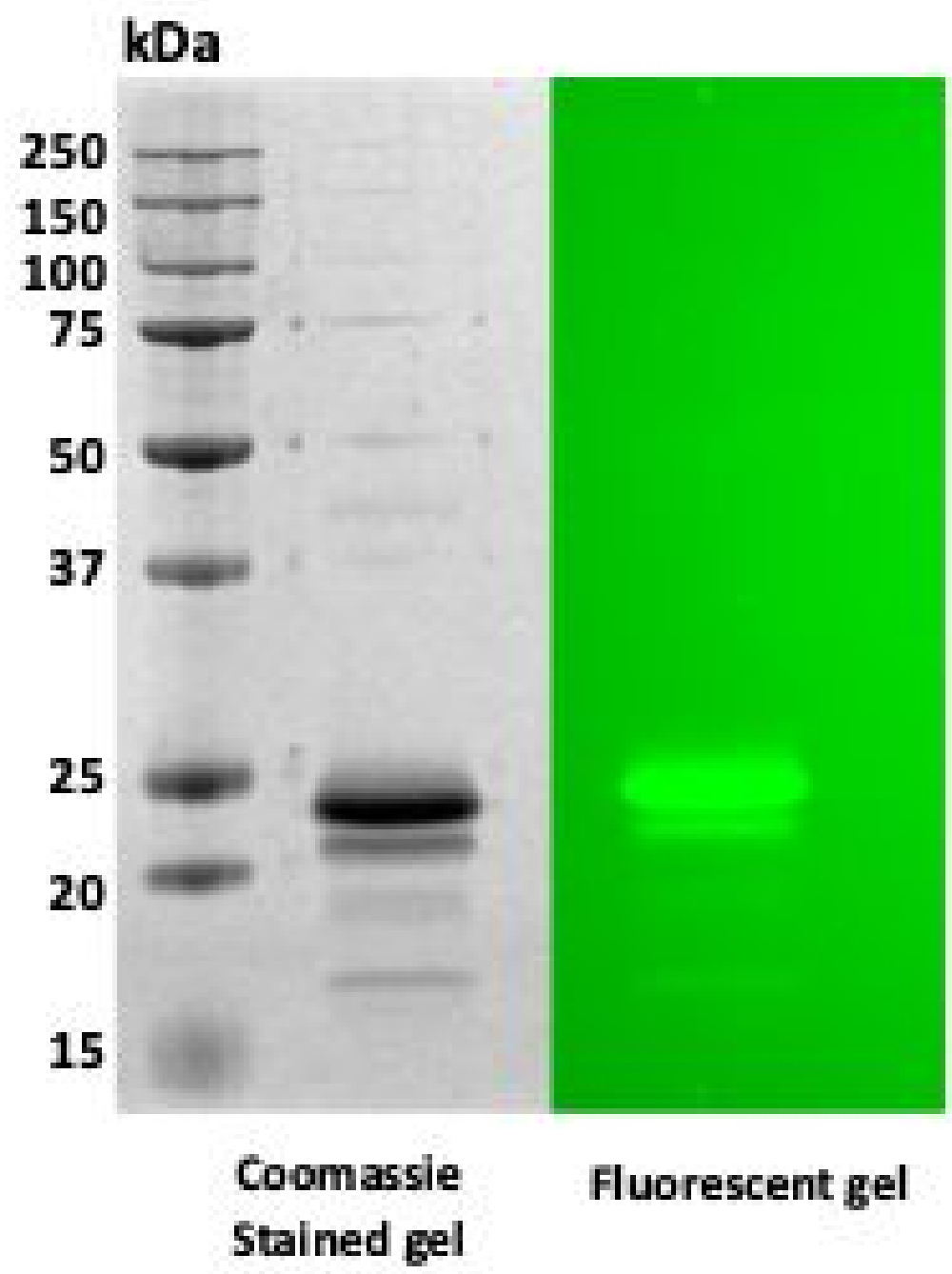

Figure S2: Electrophoretic mobility of purified W99 7-HCAA recPrP. Coomassie-stained (left) and fluorescent visualization (right) of the same SDS-PAGE polyacrylamide gel showing the purified W99 7-HCAA recPrP protein. Acrylamide gel stained with ReadyBlue® Protein Gel Stain.

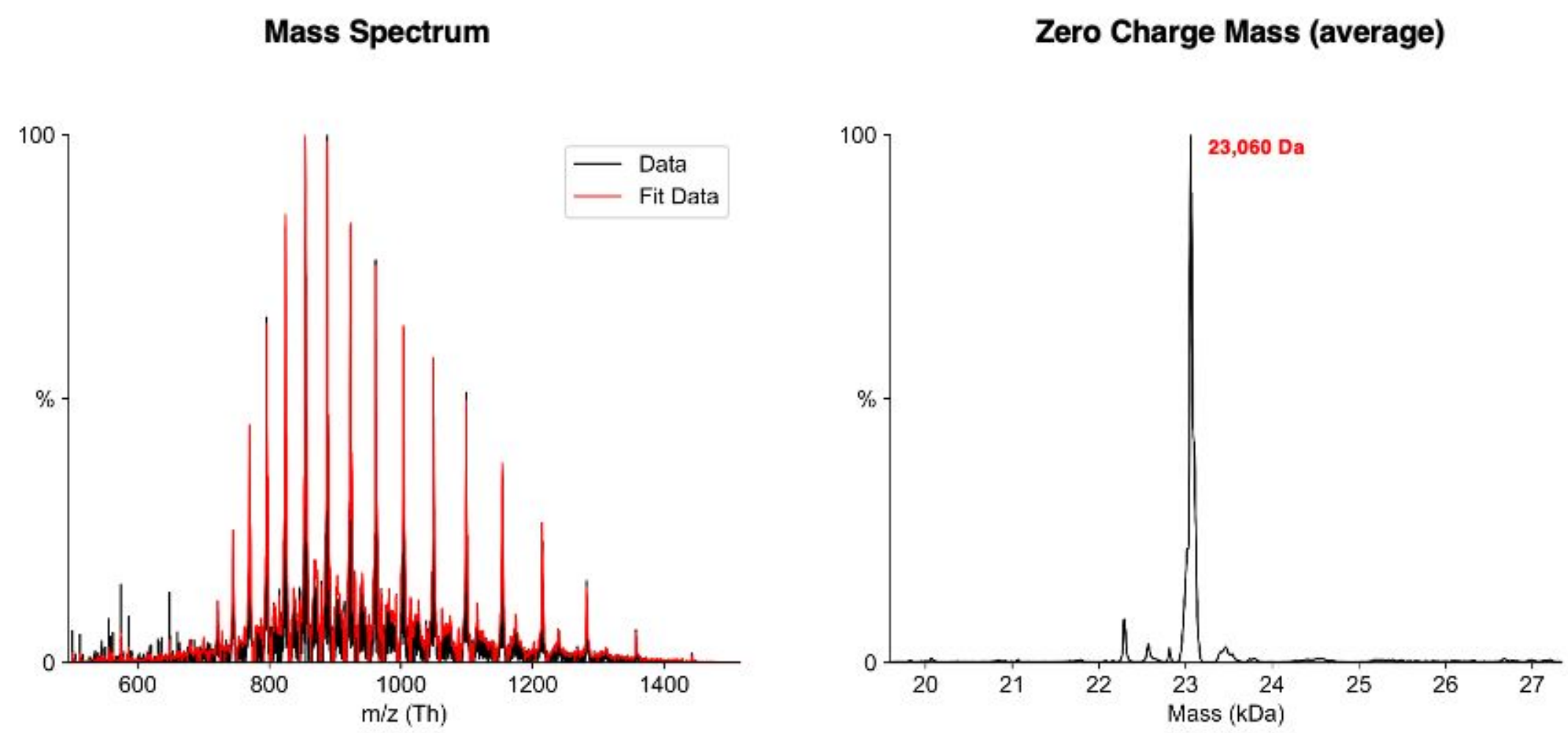

Figure S3. Mass spectrometric characterization of W99 7-HCAA recPrP. (A) Representative electrospray ionization mass spectrum showing the charge-state distribution of the purified protein. (B) Deconvoluted mass spectrum revealing a major species with a molecular mass of 23060 Da, consistent with the expected mass of the full-length protein containing the site-specifically incorporated 7-HCAA residue (23060.39 Da).

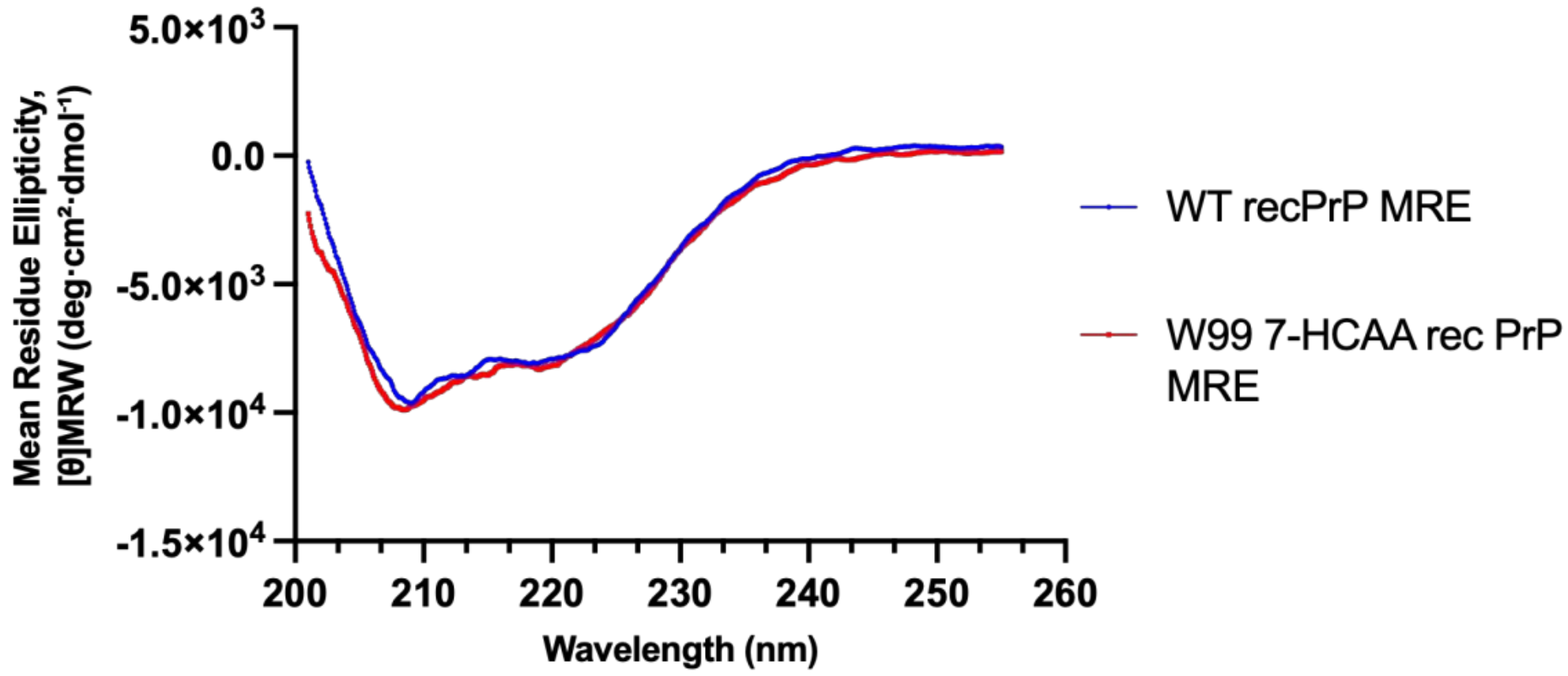

Figure S4. Circular dichroism (CD) spectrum of W99 7-HCAA recPrP and WT recPrP. Purified proteins were each resuspended in water to 0.20 mg/mL

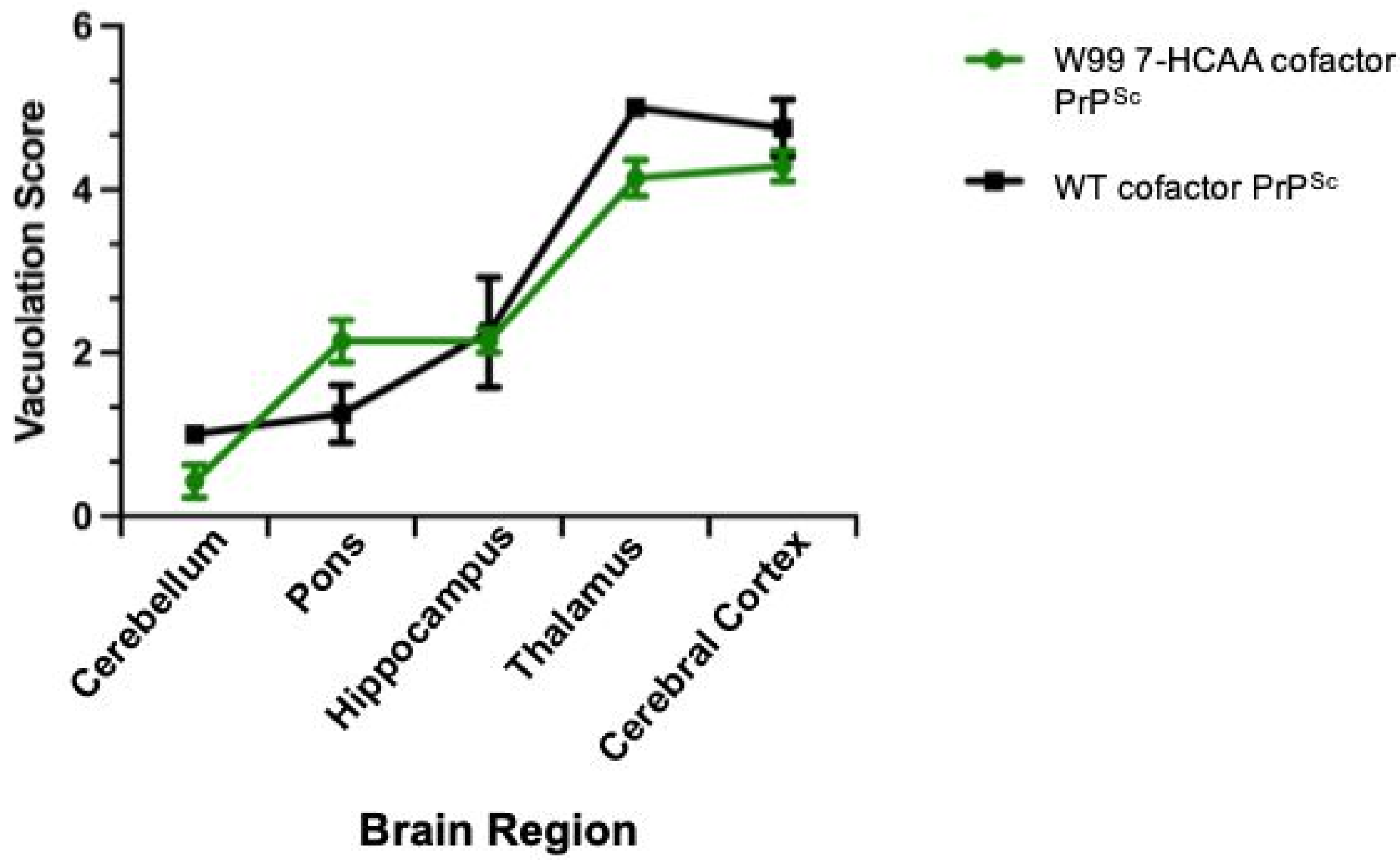

Figure S5. Regional neuropathology of kiBV M109 PrP mice inoculated with W99 7-HCAA cofactor PrP^{Sc} and WT cofactor PrP^{Sc}. Profiles of vacuolation scores of animals inoculated with either W99 7-HCAA cofactor PrP^{Sc} (green circles) or WT cofactor PrP^{Sc} (black squares). Mean values ± SEM are shown.

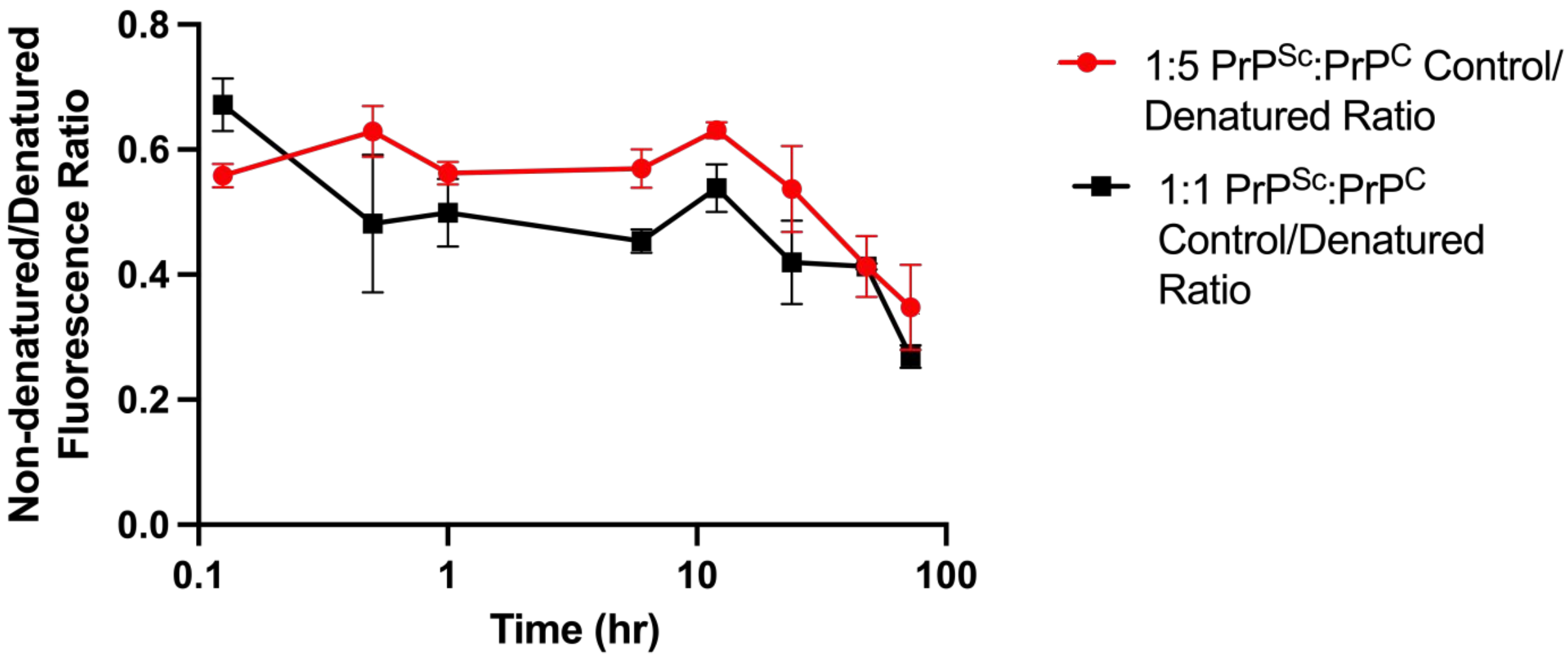

Figure S6. Time-dependent changes in the non-denatured/denatured fluorescence ratio during PrP^{Sc} formation. The ratio of non-denatured to denatured fluorescence was determined for W99 7-HCAA PrP^{Sc} propagation reactions using the standard reaction concentration (1:5 PrP^{Sc}:PrP^{C}) and a 1:1 PrP^{Sc}:PrP^{C} reaction. At each time point, aliquots were removed directly from the propagation reaction and divided into two equal portions. One aliquot was measured under non-denaturing conditions following the addition of control buffer, while the second aliquot was denatured with denaturing buffers prior to fluorescence measurement. Fluorescence values were corrected by subtraction of the corresponding buffer blank and normalized to a 7-HCAA standard included in each experiment to account for measurement-to-measurement variability. The non-denatured/denatured fluorescence ratio was calculated independently for each replicate, and the plotted values represent the mean ± SD. The x-axis is displayed on a logarithmic (base 10) scale to improve visualization of early propagation time points.

Table S1. Integrated fluorescence intensity of PrP samples. Area under the fluorescence emission curve (AUC) was calculated from emission spectra collected between 400 and 500 nm following excitation at 360 nm. Values are shown for native and denatured W99-7-HCAA cofactor and protein-only PrP^{Sc}, as well as corresponding PrP^{C} cocktail controls and WT protein-only PrP^{Sc}.

Sample	AUC
W99 7-HCAA Cofactor PrP^{Sc}	3,060,432
W99 7-HCAA Protein-Only PrP^{Sc}	7,290,563
WT Protein-Only PrP^{Sc}	734,195
W99 7-HCAA Cofactor Cocktail (PrP^{C})	10,318,277
W99 7-HCAA Protein-Only Cocktail (PrP^{C})	17,775,625
Denatured W99 7-HCAA Cofactor PrP^{Sc}	31,394,313
Denatured W99 7-HCAA Protein-Only PrP^{Sc}	29,701,020
Denatured WT Protein-Only PrP^{Sc}	5,574,647
Denatured W99 7-HCAA Cofactor Cocktail (PrP^{C})	23,921,357
Denatured W99 7-HCAA Protein-Only Cocktail (PrP^{C})	23,259,588
